# Development of a Direct *in vitro* Plant Regeneration Protocol From *Cannabis sativa* L. Seedling Explants: Developmental Morphology of Shoot Regeneration and Ploidy Level of Regenerated Plants

**DOI:** 10.3389/fpls.2020.00645

**Published:** 2020-05-27

**Authors:** Alberto Galán-Ávila, Edgar García-Fortea, Jaime Prohens, Francisco Javier Herraiz

**Affiliations:** ^1^Ploidy and Genomics S.L., Centro Europeo de Empresas Innovadoras de Valencia, Parc Tecnològic, Valencia, Spain; ^2^Instituto Universitario de Conservación y Mejora de la Agrodiversidad Valenciana, Universitat Politècnica de València, Valencia, Spain

**Keywords:** cannabinoids, hemp, hypocotyl, micropropagation, polyploidization, polysomaty, shoot organogenesis

## Abstract

*In vitro* shoot regeneration can efficiently contribute to the improvement of recalcitrant *Cannabis sativa* L. We aimed at developing a highly efficient protocol for *in vitro* direct regeneration of *C. sativa* plants from different explants (cotyledon, hypocotyl, and true leaf) from seedlings of monoecious *C. sativa* short-day varieties Ferimon, Felina32, Fedora17, and USO31, together with dioecious neutral-day variety Finola. Ten regeneration media, including already published protocols, and self-designed combinations of plant growth regulators were tested. The developmental morphology since germination of seeds to the development of rooted plantlets was followed. Additionally, the ploidy level of explants and *in vitro* regenerants was analyzed. We concluded that hypocotyl is the best explant for *in vitro* direct regeneration of *C. sativa* plants with 49.45% of responding explants, while cotyledon and true leaf had a poor response with, respectively, 4.70 and 0.42% of explants developing plantlets. In terms of shoot regeneration, we found significant differences among the culture media evaluated and the varieties studied. Overall, the best regeneration media were ZEA^RIB^ 2.0 (mg/L) and ZEA^RIB^ 1.0 (mg/L) + NAA 0.02 (mg/L) with 66.67% of responding hypocotyls. Amazingly, hypocotyls cultured in medium without plant growth regulators showed an excellent response (61.54% of responding hypocotyls) and spontaneous rooting of regenerants (17.94%). *In vitro* regenerated plants were acclimatized just 6 weeks after culture initiation. The developmental morphology study suggests that regenerated shoots originate from pericycle cells adjacent to xylem poles. Polysomaty was detected in hypocotyls and cotyledons of all varieties studied, and diploid (>80%) and mixoploid (with diploid and tetraploid cells) plants were regenerated. Our protocol allows a high shoot organogenesis efficiency in different *C. sativa* varieties. The fact that a significant percentage of plants are mixoploid may provide an alternative way to develop polyploids in *C. sativa*. Our results show that direct *in vitro* regeneration may make a significant contribution to the development of improved *C. sativa* materials for medical applications.

## Introduction

*Cannabis sativa* L. (2*n* = 2× = 20) is a dicotyledonous species belonging to Cannabaceae family used for multiple purposes (fiber, oil, edible seeds, medicinal, drug) which comprises short and neutral-day varieties. Among its different applications, its use in medicine, derived from its content in cannabinoids (Cascio et al., 2017), is raising an increasing interest. Among cannabinoids, Δ9-tetrahydrocannabinol (THC) and cannabidiol (CBD) are generally the most abundant in the plant (Andre et al., 2016). Recent research has reported many cannabinoid pharmacodynamic and pharmacokinetic properties, expanding the potential use of cannabinoids in medical therapies (Urits et al., 2019), and promoting the development of cannabis improved varieties with specific biochemical profiles. In this respect, *in vitro* culture is a useful tool that has been employed to complement cannabis conventional breeding through large-scale micropropagation of selected elite clones (Lata et al., 2017), development of polyploid varieties with enhanced levels of secondary metabolites (Mansouri and Bagheri, 2017; Parsons et al., 2019) or genetic transformation of non-regenerating tissues (Feeney and Punja, 2003, 2015, 2017; Wahby et al., 2013, 2017). However, there is still a lack of an *in vitro* regeneration protocol efficient in the broad range of genetically diverse materials in the species.

In this respect, plant regeneration is an essential step for most *in vitro* culture techniques employed in plant breeding. High rates of *in vitro* plant regeneration from already developed apical and axillary meristems of the plant (Richez-Dumanois et al., 1986; Lata et al., 2009, 2016a,b), young leaves (Lata et al., 2010) and cotyledons (Chaohua et al., 2016) have already been reported in *C. sativa*. However, several studies point out to a high level of recalcitrance of *in vitro* shoot regeneration from different tissues such as maturing bracts, anther-calyx complexes and vegetative leaves (Hemphill et al., 1978), leaves, hypocotyls, cotyledons, and roots (Mandolino and Ranalli, 1999), young leaves, petioles, internodes, and axillary buds (Lusarkiewicz-Jarzina et al., 2005), roots, leaves and stems (Plawuszewski et al., 2006), cotyledons, stems and roots (Wielgus et al., 2008), cotyledons and epicotyls (Movahedi et al., 2015), leaves and hypocotyls (Movahedi et al., 2016a, b), hemp transformed roots (Wahby et al., 2017), and hypocotyl segments (Smýkalová et al., 2019). Therefore, the low regeneration efficiency of published *in vitro* plant regeneration protocols for this species, and its wide variation among explant types and varieties represent a major bottleneck for the application of *in vitro* tissue culture to the improvement of *C. sativa*. Moreover, in most of the aforementioned publications, a small number of varieties were evaluated, which not represent all subspecies and reproductive systems present in the species. In addition, in the vast majority of these studies, when shoot regeneration was successful, it developed in an indirect way through a previous phase of callus formation, which may compromise the genetic fidelity of regenerants with respect to the donor plant (Evans and Bravo, 1986; Ramírez-Mosqueda and Iglesias-Andreu, 2015). Finally, a common feature that can be inferred from previously mentioned studies is that development of *in vitro* shoot organogenesis in this species requires addition of plant growth regulators to the culture medium.

Considering the above mentioned facts, the aim of this study was focused on the development of a highly efficient species-specific protocol for *in vitro* direct regeneration of *C. sativa* plants. For this, we evaluated different explants such as cotyledon, hypocotyl and true leaf coming from seedlings of four monoecious short-day varieties plus a dioecious hemp neutral-day variety, which were employed as donor plants. Explants were cultured on media with different plant growth regulators and hormonal concentrations obtained from already published protocols, together with self-designed combinations of plant growth regulators. The developmental morphology process of *in vitro* shoot organogenesis from cotyledons, hypocotyls and true leaves was followed and registered with images, and the duration of each of the developmental stages of organogenesis was also recorded. Additionally, due to the lack of studies concerning polysomaty in this species and its potential usefulness to obtain polyploid plants as it has occurred in other species like tomato (Van den Bulk et al., 1990), cucumber (Colijn-Hooymans et al., 1994), or melon (Ren et al., 2013), and more recently in eggplant (García-Fortea et al., 2020), authors not only studied the regenerative capacity of cotyledons, hypocotyls and true leaves, but also analyzed the ploidy level of these explants together with that of the *in vitro* regenerants.

## Materials and Methods

### Plant Material and Growth Conditions

Seeds from monoecious *C. sativa* short-day varieties Ferimon, Felina32, Fedora17, and USO31, together with seeds from dioecious neutral-day variety Finola were surface sterilized in 75% (v/v) ethanol during 2 min and 30 s, followed by immersion in 30 g/L of NaClO with 0.1% (v/v) of Tween 20 during 25 min, and finally washed three times in autoclaved deionized water. Once sterilized, seeds were germinated in 9 cm diameter plastic Petri dishes containing previously autoclaved germination medium which composition was ½ MS basal salts and vitamins (Murashige and Skoog, 1962) + 1.5% (w/v) sucrose + 3.5 g/L Gelrite^®^ with a pH value of 5.8. After germination, cotyledons, hypocotyls and true leaves dissected from 7-days-old seedlings were employed as explants. In this species, this stage of seedling development is equivalent to the phenological growth stage coded in *Biologische Bundesanstalt, Bundessortenamt*, and *CHemical industry* (BBCH-scale) by number 11 (Mishchenko et al., 2017). Explants were cultured in the different media described in Table 1. Seedlings and explants were grown under controlled conditions at 22°C ± 1°C and 60% ± 1% relative humidity. Photoperiod consisted of 16 h of light and 8 h of dark. Light was provided by Light Emitting Diode (LED) tubes of 18 W and a color temperature of 6000 K, which provided 6,010 lux and 90.15 μmol m^–2^ s^–1^. Explants producing shoots and roots, and number of shoots developed on each of responding explants were counted periodically during 2 weeks of culture. After that time, *in vitro* regenerants were subcultured individually to glass-tubes of 2.5 cm of diameter and 15 cm long, containing the same medium in which shoots were generated.

**TABLE 1:** Media tested for *in vitro* shoot induction from cotyledons, hypocotyls and true leaves of *C. sativa*, including plant growth regulators composition and their respective concentrations.

**Medium**	**Plant growth regulators (mg/L)**	**References**
0	Without plant growth regulators	–
1	TDZ (0.4) + NAA (0.2)	Chaohua et al., 2016
2	BAP (2.0) + IBA (0.5)	Movahedi et al., 2015
3	BAP (0.5) + 2,4-D (0.1)	Movahedi et al., 2016a
4	ZEA^RIB^ (2.0)	García-Fortea et al., 2020
5	BAP (1.0) + NAA (0.02)	–
6	BAP^RIB^ (1.0) + NAA (0.02)	–
7	TDZ (1.0) + NAA (0.02)	–
8	4-CPPU (1.0) + NAA (0.02)	–
9	ZEA^RIB^ (1.0) + NAA (0.02)	–

When roots were visible, spontaneously-rooted plants were cultured in pots (2 L) with fertilized commercial substrate composed of a mixture of black peat, granulated peat moss and perlite, with a pH value of 6 and a conductivity of 1 mS/cm. Previously, gelled medium was carefully washed from roots. After transplant and during the whole process of acclimatization, the substrate was maintained slightly moist and, twice per day (early in the morning and in late afternoon), regenerants received foliar pulverization with water. To avoid desiccation, the small plants were covered with plastic vessels and were progressively exposed to the environmental humidity. Until complete acclimatization, plants were grown under identical conditions of temperature, photoperiod and light as described above. Plants employed in this study were grown under license for the cultivation of *C. sativa* for research purposes, issued by the spanish Ministry of Health, Social Services and Equality via Spanish Agency of Medicines and Health Products (Agencia Española de Medicamentos y Productos Sanitarios or AEMPS) to Ploidy and Genomics Ltd.

### *In vitro* Shoot Organogenesis Experiments

In order to promote *in vitro* shoot organogenesis in *C. sativa*, cotyledons, hypocotyls and true leaves dissected from 7-days-old seedlings were cultured in germination medium with the same composition as described above, except for the addition of different plant growth regulators. As a part of this study, we aimed at evaluating with our own genotypes the efficiency of different *in vitro* shoot regeneration published protocols developed for *C. sativa*. Therefore, we selected studies in which different explants, cytokinins and auxins and their respective concentrations were successfully tested. In this way, we tested the media used in a study regarding the regenerative capacity of cotyledons through addition of thidiazuron (TDZ) and α-naphthaleneacetic acid (NAA) to the culture medium (Chaohua et al., 2016), one work concerning *in vitro* plant regeneration from cotyledons and epicotyls by means of 6-benzylaminopurine (BAP) and indole-3-butyric acid (IBA) (Movahedi et al., 2015), and another one from leaves and hypocotyls through BAP and 2,4-dichlorophenoxyacetic acid (2,4-D) (Movahedi et al., 2016a). Additionally, it was added to our schedule an effective and newly released protocol developed for eggplant in which the use of zeatin riboside (ZEA^RIB^), provided good results not only in terms of shoot organogenesis, but also in polyploidization of regenerants (García-Fortea et al., 2020).

Finally, as it is known that root and shoot development depends on cytokinin:auxin ratio and that high levels of cytokinin supports shoot formation (Skoog and Miller, 1957; Su et al., 2011), we tested the effect of media with a cytokinin concentration 50-fold higher than the auxin level, together with different adenine and phenylurea derivatives like BAP, 6-benzylaminopurine riboside (BAP^RIB^), TDZ, forchlorfenuron (4-CPPU) and ZEA^RIB^ plus NAA, an auxin commonly employed in protocols for *in vitro* regeneration of shoots (Plawuszewski et al., 2006; Wielgus et al., 2008; Lata et al., 2010; Chaohua et al., 2016) and *in vitro* rooting of *C. sativa* (Lusarkiewicz-Jarzina et al., 2005; Wang et al., 2009; Movahedi et al., 2016a; Parsons et al., 2019). The different hormonal combinations present in the different shoot induction media evaluated in this work, are detailed in Table 1.

### Developmental Morphology of the *in vitro* Regeneration Process

The whole developmental process of *in vitro* shoot organogenesis, since germination of seeds until acclimatization of plants was followed and registered with images. The time needed for each of the different developmental stages was recorded. High resolution images of the different developmental stages were recorded with an Optika^®^ SZN-6 (OPTIKA S.r.l., Ponteranica, Italy) laboratory stereozoom microscope equipped with an Optika^®^ C-HP (OPTIKA S.r.l.) digital camera.

### Determination of Ploidy Level of Explants and *in vitro* Regenerants

Ploidy level of cotyledons, hypocotyls and leaves from *in vitro* grown 7-days-old seedlings was evaluated to verify their polysomatic pattern. The four monoecious short-day varieties Ferimon, Felina32, Fedora17, and USO31, together with dioecious neutral-day variety Finola were analyzed in this experiment. Three seedlings coming from each variety were employed for this assay. On the other hand, young leaves from *in vitro* regenerated plants were also examined. Ploidy level of 35 *in vitro* regenerants (17 from cotyledons, 15 from hypocotyls and 3 from leaves) was determined. Cell nuclei of explants dissected were mechanically isolated. Sections of approximately 0.5 cm^2^ were chopped with a razor blade in a 6 cm diameter glass Petri dish containing 0.5 ml lysis buffer LB01 (pH 7.5) (Dpooležel et al., 1989), and incubated for 5 min. Subsequently, the suspension containing nuclei and cell fragments was filtered using a 30 μm CellTrics filter (Sysmex, Sant Just Desvern, Spain). The nuclei in the filtrate were stained with CyStain UV Ploidy (Sysmex) and incubated for 5 min. The fluorescence intensity of the homogenate was measured using a CyFlow Ploidy Analyser Sysmex Partec GmbH, analyzing at least 4,000 nuclei for each sample. Young leaves of diploid plants from all varieties studied were used as control. A diploid control peak was established at 50 points of the arbitrary intensity value of the fluorescence in the histogram. By comparison with this peak, the ploidy of the other tissues evaluated was checked.

### Data Analyses

In order to develop a highly efficient protocol for *in vitro* direct regeneration of *C. sati*va plants, we analyzed statistically the effect of different factors such as genotype, explant and culture medium on *in vitro* shoot organogenesis. For each factor, the mean of responding explants was expressed as a percentage (±SE) relative to the total amount of cultured explants. For varieties and media with the best shoot induction rates identified in this study, also the number of shoots per responding explant were statistically evaluated. Data recorded until the second week of culture were employed for the statistical analysis. Additionally, the effect of the explant factor on the ploidy level of *in vitro* regenerants was statistically determined. For each explant, the mean of diploid and mixoploid regenerants was expressed as a percentage (±SE) relative to the total amount of plants submitted to flow cytometry analysis. While the factor genotype was represented through four monoecious short-day varieties (Ferimon, Felina32, Fedora17, and USO31) plus a dioecious hemp neutral-day variety (Finola), the explants evaluated in this assay were cotyledons, hypocotyls, and true leaves coming from 7-days-old seedlings. The 10 different media described in Table 1 constituted the culture medium factor. Each factor was analyzed using at least five biological replicates. Each biological replicate consisted of a Petri dish containing 3 explants coming from 3 different seedlings of the same variety in the case of hypocotyls, and 6 explants coming from 3 different seedlings of the same variety in the case of cotyledons and true leaves. A total of 2,463 explants were employed in this work (1,000 from cotyledons, 275 from hypocotyls and 1,188 from true leaves). Independence among variables (distribution-plot test), homoscedasticity (Bartlett’s test), and normality (Shapiro–Wilk test) were evaluated for the data coming from the experiments. Given that none of the three criteria were met, Kruskal-Wallis non-parametric test followed by pairwise Wilcoxon test (*p* < 0.05) was used to evaluate statistical significance of differences between factors. Statistical analysis was carried out using R software (Ihaka and Gentleman, 1996).

## Results

### Effect of Genotype, Explant and Medium on *in vitro* Shoot Organogenesis of *C. sativa*

Shoot *in vitro* regeneration was observed in all *C. sativa* varieties, explant types and media tested, resulting in a total of 255 *in vitro* regenerated shoots, although significant differences (*p* < 0.05) between the different levels of the three main factors were observed (Table 2). Regarding the factor explant and its effect on the percentage of explants developing shoots, significant differences were detected between cotyledons, hypocotyls, and true leaves. On average, hypocotyl showed the best response in terms of direct plant regeneration, reaching 49.45% of explants with shoot formation, followed by cotyledon with 4.70% and true leaf with 0.42% (Table 2). Also, significant differences between varieties were observed. USO31 was the best variety evaluated in this experiment, with 12.32% of their explants exhibiting shoot organogenesis, while the variety with a lower percentage of regeneration was Finola with only 4.62% (Table 2). Finally, only medium 1 (TDZ 0.4 mg/L + NAA 0.2 mg/L) showed a significantly higher capacity in promoting shoot organogenesis, achieving an average of 15.78% of induction rate (Table 2).

**TABLE 2:** Effect of genotype, explant and medium on direct *in vitro* shoot organogenesis rate of different explants from *C. sativa*.

	**Responding**		
**Factor**	**explants (%)**	**Significance^a^**	**n**
**Variety**			
Ferimon	6.23 ± 1.06	bc	514
Felina32	7.37 ± 1.03	bc	638
Fedora17	8.55 ± 1.36	ab	421
USO31	12.32 ± 1.61	a	414
Finola	4.62 ± 0.96	c	476
**Explant**			
Cotyledon	4.70 ± 0.66	b	1000
Hypocotyl	49.45 ± 3.02	a	275
Leaf	0.42 ± 0.18	c	1188
**Medium (mg/L)**			
0 → Without plant growth regulators	6.81 ± 1.24	b	411
1 → TDZ 0.4 + NAA 0.2	15.78 ± 1.79	a	412
2 → BAP 2+ IBA 0.5	4.61 ± 1.25	b	282
3 → BAP 0.5 + 2,4-D 0.1	6.42 ± 1.50	b	265
4 → ZEA^RIB^ 2	6.01 ± 1.76	b	183
5 → BAP 1 + NAA 0.02	5.50 ± 1.54	b	218
6 → BAP^RIB^ 1 + NAA 0.02	6.71 ± 1.95	b	164
7 → TDZ 1 + NAA 0.02	4.37 ± 1.51	b	183
8 → 4-CPPU 1 + NAA 0.02	8.67 ± 2.30	b	150
9 → ZEA^RIB^ 1 + NAA 0.02	5.13 ± 1.58	b	195

*Mean of responding explants (%), significance and sample size (n) are presented in different columns. For each factor, mean of responding explants is expressed as a percentage (±SE) relative to the total amount of cultured explants. ^a^Different letters among the levels of each of the three factors indicate significant differences between them (*p* < 0.05) according to non-parametric Kruskal-Wallis and pairwise Wilcoxon tests.*

Since true leaves displayed a weak capacity to induce direct shoot organogenesis, and in order to eliminate the negative effect that they were adding to the variety and medium factors, we analyzed separately data from cotyledons (Table 3) and hypocotyls (Table 4). Regarding cotyledons, significant differences were observed among varieties. USO31 reached the highest rate of shoot organogenesis with 9.29% (Table 3), while Felina32 exhibited the lowest shoot induction rate with 2.10% (Table 3). With respect to the factor medium, medium 1 (TDZ 0.4 mg/L + NAA 0.2 mg/L) was the best, achieving the highest shoot induction rate with a 22.32% of responding explants (Table 3). Medium 0 (without plant growth regulators) and number 9 (ZEA^RIB^ 1 mg/L + NAA 0.02 mg/L) were the worst treatments, without any explant showing response in terms of shoot organogenesis (Table 3).

**TABLE 3:** Effect of genotype and medium on direct *in vitro* shoot organogenesis rate of cotyledons from *C. sativa*.

**Factor**	**Responding explants (%)**	**Significance^a^**	**n**	**Shoots per responding explant**	**Significance^a^**	**n**
**Variety**						
Ferimon	5.50 ± 1.61	ab	200	1.09 ± 0.09	a	11
Felina32	2.10 ± 0.84	c	286	1.17 ± 0.17	*	6
Fedora17	6.67 ± 1.86	ab	180	1.42 ± 0.15	a	12
USO31	9.29 ± 2.46	a	140	1.00 ± 0.00	a	13
Finola	2.58 ± 1.14	bc	194	1.00 ± 0.00	*	5
**Medium (mg/L)**						
0 → Without plant growth regulators	0.00 ± 0.00	d	234	–	*	0
1 → TDZ 0.4 + NAA 0.2	22.32 ± 3.95	a	112	1.28 ± 0.09	a	25
2 → BAP 2 + IBA 0.5	1.85 ± 1.30	c	108	1.00 ± 0.00	*	2
3 → BAP 0.5 + 2,4-D 0.1	5.56 ± 2.42	bc	90	1.00 ± 0.00	*	5
4 → ZEA^RIB^ 2	1.28 ± 1.28	cd	78	1.00	*	1
5 → BAP 1 + NAA 0.02	1.92 ± 1.35	c	104	1.00 ± 0.00	*	2
6 → BAP^RIB^ 1 + NAA 0.02	6.25 ± 3.04	bc	64	1.00 ± 0.00	*	4
7 → TDZ 1 + NAA 0.02	2.56 ± 1.80	c	78	1.00 ± 0.00	*	2
8 → 4-CPPU 1 +NAA 0.02	14.29 ± 5.46	ab	42	1.00 ± 0.00	a	6
9 → ZEA^RIB^ 1 + NAA 0.02	0.00 ± 0.00	d	90	–	*	0

Mean of responding explants (%), significance and sample size (*n*) are presented in different columns. For each factor, mean of responding explants is expressed as a percentage (±SE) relative to the total amount of cultured explants. This table also includes the effect of genotype and medium on the number of shoots per responding cotyledon of *C. sativa*. Mean number of shoots per responding explant (±SE), significance and sample size (*n*) are presented in different columns. Shoots per responding explant from varieties and media with the best shoot induction rates are statistically compared. ^*a*^Different letters among the levels of each of the two factors indicate significant differences between them (*p* < 0.05) according to non-parametric Kruskal-Wallis and pairwise Wilcoxon tests. * Not analyzed statistically.

**TABLE 4:** Effect of genotype and medium on direct *in vitro* shoot organogenesis rate of hypocotyls from *C. sativa*.

**Factor**	**Responding explants (%)**	**Significance^a^**	**n**	**Shoots per responding explant**	**Significance^a^**	**n**
**Variety**						
Ferimon	32.26 ± 5.98	c	62	1.25 ± 0.12	*	20
Felina32	62.50 ± 6.09	ab	64	1.20 ± 0.06	b	40
Fedora17	44.90 ± 7.17	bc	49	1.50 ± 0.13	*	22
USO31	71.15 ± 6.34	a	52	1.72 ± 0.12	a	37
Finola	35.42 ± 6.97	c	48	1.59 ± 0.12	*	17
**Medium (mg/L)**						
0 → Without plant growth regulators	61.54 ± 7.89	ab	39	1.54 ± 0.12	a	24
1 → TDZ 0.4 + NAA 0.2	54.17 ± 5.91	ab	72	1.49 ± 0.11	a	39
2 → BAP 2 + IBA 0.5	36.67 ± 8.94	c	30	1.27 ± 0.14	*	11
3 → BAP 0.5 + 2,4-D 0.1	38.71 ± 8.89	c	31	1.33 ± 0.19	*	12
4 → ZEA^RIB^ 2	66.67 ± 12.59	a	15	1.60 ± 0.16	a	10
5 → BAP 1 + NAA 0.02	41.67 ± 10.27	bc	24	1.40 ± 0.16	*	10
6 → BAP^RIB^ 1 + NAA 0.02	43.75 ± 12.80	bc	16	1.29 ± 0.18	*	7
7 → TDZ 1 + NAA 0.02	40.00 ± 13.09	c	15	1.50 ± 0.22	*	6
8 → 4-CPPU 1 + NAA 0.02	38.89 ± 11.82	c	18	1.43 ± 0.20	*	7
9 → ZEA^RIB^ 1 + NAA 0.02	66.67 ± 12.59	a	15	1.30 ± 0.15	a	10

Mean of responding explants (%), significance and sample size (*n*) are presented in different columns. For each factor, mean of responding explants is expressed as a percentage (±SE) relative to the total amount of cultured explants. This table also includes the effect of genotype and medium on the number of shoots per responding hypocotyl of *C. sativa*. Mean number of shoots per responding explant (±SE), significance and sample size (*n*) are presented in different columns. Shoots per responding explant from varieties and media with the best shoot induction rates are statistically compared. ^*a*^Different letters among the levels of each of the two factors indicate significant differences between them (*p* < 0.05) according to non-parametric Kruskal-Wallis and pairwise Wilcoxon tests. * Not analyzed statistically.

Concerning hypocotyls, significant differences were identified between the different varieties and media evaluated in this experiment. Again, USO31 was the best variety evaluated, with 71.15% of its explants developing shoots (Table 4), while Finola and Ferimon were the varieties with the lowest regeneration percentages with, respectively, 35.42 and 32.26% of its explants regenerating shoots (Table 4). In relation to the effect of medium on shoot organogenesis, media number 4 (ZEA^RIB^ 2 mg/L) and number 9 (ZEA^RIB^ 1 mg/L + NAA 0.02 mg/L) resulted in the highest rate of shoot induction with 66.67% of responding explants, followed by medium 0 (without plant growth regulators) and medium 1 (TDZ 0.4 mg/L + NAA 0.2 mg/L) with, respectively, 61.54 and 54.17% of shoot organogenesis rate (Table 4).

In addition, the number of shoots developed on each of the responding explants were statistically analyzed for varieties and media with the best shoot induction rates identified in this study. In the case of cotyledons, as varieties USO31, Fedora17 and Ferimon, and media 1 (TDZ 0.4 mg/L + NAA 0.2 mg/L) and 8 (4-CPPU 1 mg/L + NAA 0.02 mg/L) gave the best shoot induction rates, their number of shoots per responding explant were statistically compared (Table 3). Although no significant differences were found between varieties and media in terms of number of shoots per responding cotyledon, Fedora17 showed the best results with 1.42 shoots per responding explant, while medium 1 (TDZ 0.4 mg/L + NAA 0.2 mg/L) reached 1.28 shoots per responding cotyledon (Table 3).

Regarding hypocotyls, since varieties USO31 and Felina32, and media 0 (without plant growth regulators), 1 (TDZ 0.4 mg/L + NAA 0.2 mg/L), 4 (ZEA^RIB^ 2 mg/L), and 9 (ZEA^RIB^ 1 mg/L + NAA 0.02 mg/L) attained the best shoot organogenesis rates, their number of shoots per responding explant were also statistically compared (Table 4). USO31 exhibited the best response in terms of number of shoots per responding hypocotyl, reaching 1.72 shoots per responding explant (Table 4). Furthermore, although no significant differences were found among media tested, medium 4 (ZEA^RIB^ 2 mg/L), closely followed by medium 0 (without plant growth regulators), were the best media evaluated in this experiment with, respectively, 1.60 and 1.54 shoots per responding hypocotyl (Table 4).

### Developmental Morphology of *in vitro* Shoot Organogenesis in *C. sativa*

**FIGURE 1:**
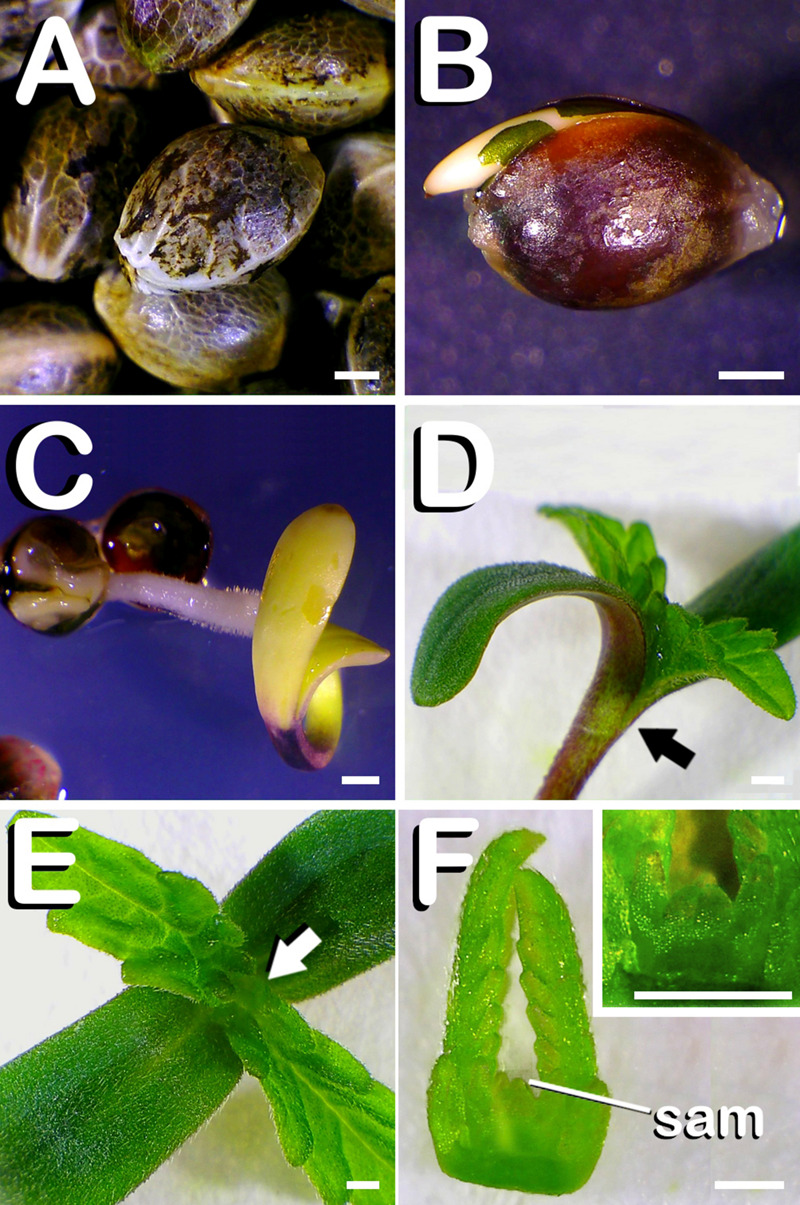
Seed germination of *C. sativa*. The different developmental stages are described as follows: **(A)** Seeds just before being sterilized. **(B)** Germinated seed 48 h. after *in vitro* sowing with the root apical meristem arising from testa. **(C)** Emerging seedling 5 days after seed plating, with testa being visible at the bottom of the image. **(D)** Seven-days-old seedling with fully expanded first pair of true leaves, which is equivalent to the phenological growth stage coded in this species by number 11 in BBCH-scale: arrow marks dissection point. **(E)** View of 7-days-old seedling allowing observation of vegetative shoot apex: arrow points shoot apex location on seedling. **(F)** Remaining vegetative shoot apex after dissection of hypocotyl, cotyledon and true leaves from 7 days-old seedling, with shoot apical meristem (SAM) highlighted on it: detail of SAM (inset in panel **F**). Scale bars: 1 mm.

First of all, seeds of the different varieties (Figure 1A) were surface sterilized. Between 24 and 48 h after being cultured in germination medium, seeds started to germinate and the apical root meristem arose from the testa (Figure 1B). Five days after *in vitro* sowing, seedlings liberated from the testa were visible while emerging (Figure 1C). On the seventh day from seed plating, the first pair of true leaves was fully expanded (Figure 1D). When seedlings arrived to this developmental stage, explants needed to continue the experiments were obtained through a clean cut across dissection point (arrow in Figure 1D). Remaining vegetative shoot apex located on the top of the seedling (arrow in Figure 1E) was discarded. As can be observed in Figure 1F, discarded shoot apex preserved the whole shoot apical meristem (SAM) (inset in Figure 1F).

**FIGURE 2:**
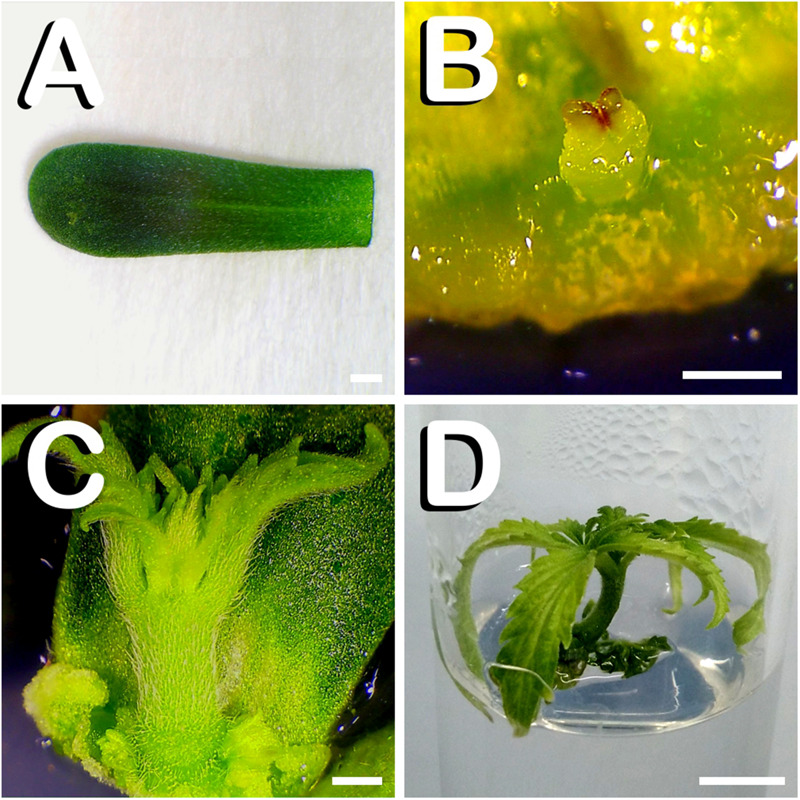
Direct *in vitro* shoot organogenesis from cotyledon leaves of *C. sativa*. The different developmental stages are described as follows: **(A)** Newly dissected cotyledon leaf from a 7-days-old hemp seedling. **(B)** Shoot primordium formation at the basal zone of cotyledon leaf after 4 days of *in vitro* culture. **(C)** Vigorous shoot arising from the lower part of cotyledon leaf 14 days after exposure to the culture medium. **(D)** Cotyledon derived plant cultured in a glass-tube 21 days after explant inoculation. Scale bars **(A–C)**: 1 mm. Scale bar **(D)**: 6 mm.

At this stage of seedling development, cotyledon leaves were easily dissected (Figure 2A). The first steps of direct shoot organogenesis were rapidly visible on the basal zone of responding cotyledons. Shoot primordia formation was centrally located at the proximal part of cotyledons after 4 days of *in vitro* culture (Figure 2B). Two weeks after explant transfer to shoot induction medium, vigorous regenerants arising from the proximal edge of cotyledons were observed (Figure 2C). At this stage of development, since regenerants reached approximately one centimeter in height and in order to avoid their contact with the Petri dish lid, *in vitro* regenerated shoots were subcultured individually in glass-tubes containing the same medium in which they were generated (Figure 2D). A total of 47 cotyledons responded to the different treatments evaluated, generating a total amount of 54 shoots.

**FIGURE 3:**
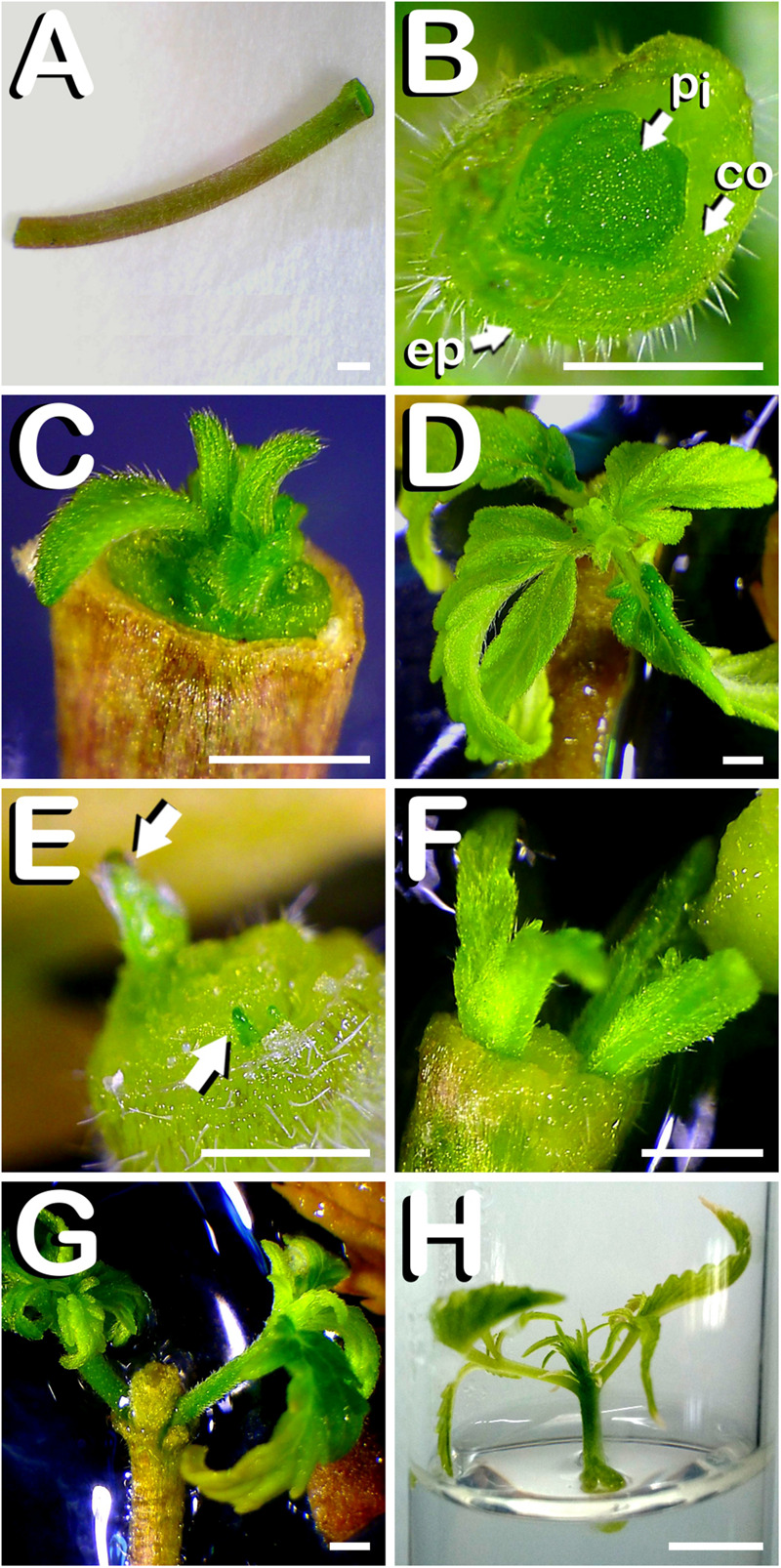
Direct *in vitro* shoot organogenesis from hypocotyls of *C. sativa*. The different developmental stages are described as follows: **(A)** Newly dissected hypocotyl from a 7-days-old hemp seedling. **(B)** Transverse section of newly dissected hemp hypocotyl revealing its different layers: ep: epidermis; co: cortex; pi: pith. **(C)** Formation of one shoot at the top of the hypocotyl after 7 days of *in vitro* culture. **(D)** Vigorous shoot arising from the upper part of hypocotyl 14 days after exposure to the culture medium. **(E)** Two primordia arising from the top of the hypocotyl after 4 days of *in vitro* culture: arrows point both primordia. **(F)** Two hypocotyl derived plants 9 days after explant inoculation. **(G)** Two hypocotyl derived regenerants ready to be subcultured 14 days after explant culture. **(H)** Hypocotyl derived plant individually grown in a glass-tube 21 days after culture initiation. Scale bars **(A–G)**: 1 mm. Scale bar (H): 6 mm.

Alternatively, hypocotyls were cut from 7-days-old seedlings. Hypocotyls employed in this experiment measured approximately one centimeter in length (Figure 3A). Transversal section of freshly dissected hypocotyls revealed its internal structure, with different tissue layers such as epidermis, cortex and pith, together with the absence of meristem traces (Figure 3B). Responding explants exhibited different direct organogenesis patterns. Some of the hypocotyls generated only one primordium on the top of the explant which was originated from the central region of the section (Figure 3C), and continued its development until becoming a robust plant after 2 weeks of culture (Figure 3D). Other explants gave rise to a couple of primordia in the periphery of the organ and on opposite sides (arrows in Figure 3E). In this last case, there were situations in which development between both primordia was asynchronous, while in other cases, growth and height of both regenerants was coincident, as can be observed in Figure 3F. In the case embodied in Figure 3G, both *in vitro* regenerants reached approximately one centimeter in height 2 weeks after culture initiation, while an overgrowth of the pith (located in the middle of the explant) was also observed. At this stage of organogenic development, shoots were detached from hypocotyls and individually cultured in glass-tubes containing the same medium in which they were generated (Figure 3H). A total of 136 hypocotyls responded to the different treatments evaluated, producing a total amount of 196 shoots.

**FIGURE 4:**
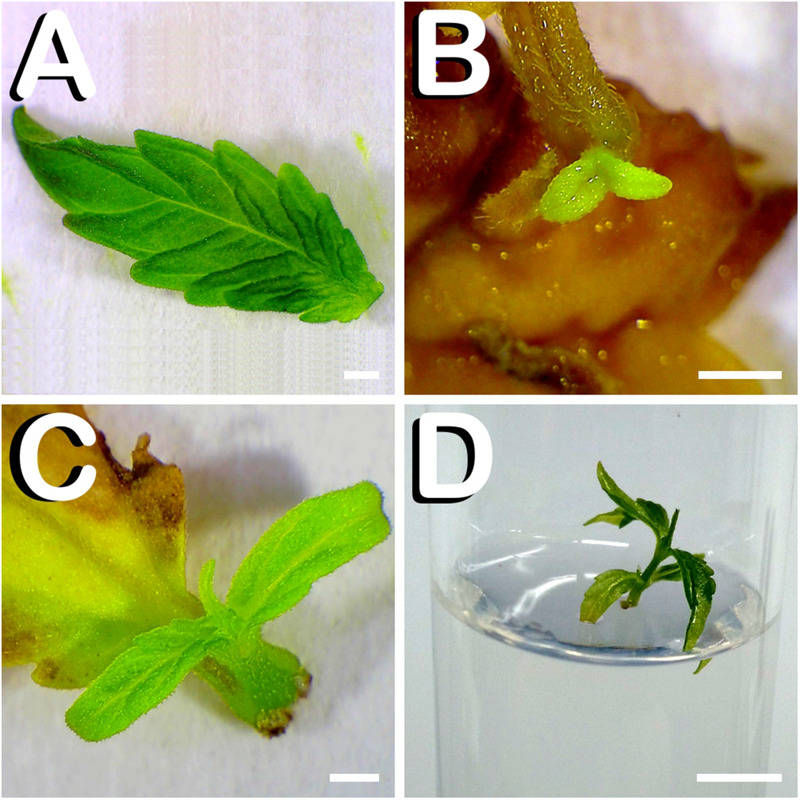
Direct *in vitro* shoot organogenesis from true leaves of *C. sativa*. The different developmental stages are described as follows: **(A)** Newly dissected leaf from a 7-days-old hemp seedling. **(B)** Formation of one primordium from leaf-petiole transition zone 1 week after culture initiation. **(C)** Two-week-old plantlet of approximately one centimeter in height ready for subculture. **(D)** Leaf derived plant individually grown in a glass-tube 21 days after culture initiation. Scale bars **(A–C)**: 1 mm. Scale bar **(D)**: 6 mm.

Finally, the regenerative capacity of the first pair of true leaves from 7-days-old seedlings was also studied. For this, each leaf was carefully dissected (Figure 4A) and cultured in the different media evaluated in this experiment. When *in vitro* plant regeneration occurred, primordia arose always from the base of leaves, specifically from the petiole fragment attached to the leaf, as is the case of Figure 4B, where a small plantlet arising from the leaf-petiole transition zone can be seen 1 week after culture. Shoot development continued until regenerated plants reached approximately one centimeter in height (Figure 4C). Fourteen days after culture initiation, successful excision of shootlets was performed and regenerated plants were individually subcultured in glass-tubes containing the same medium in which they were generated (Figure 4D). Only five leaves responded to any of the different treatments evaluated, generating a total amount of five shoots.

### Rooting of Explants and Spontaneous Rooting of Hypocotyl Derived Plants

**FIGURE 5:**
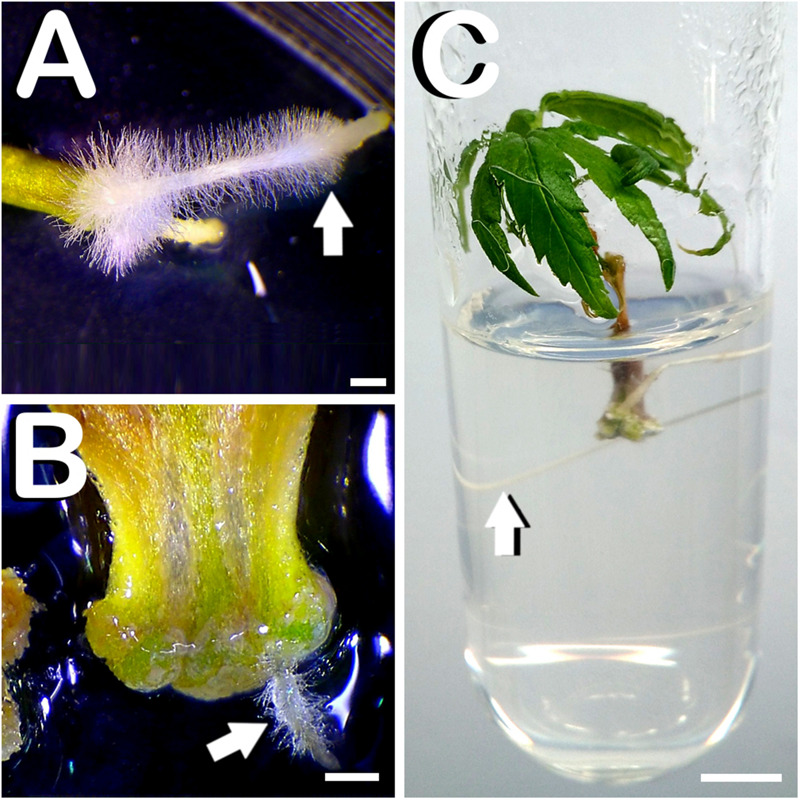
Rooting of explants and spontaneous rooting of hypocotyl derived plants of *C. sativa*. **(A)** Vigorous root with radicular hairs emerging from the basal zone of the hypocotyl 2 weeks after culture initiation (arrow). **(B)** Small root with root hairs arising from the lower part of the cotyledon after 14 days of *in vitro* culture (arrow). **(C)** Spontaneously rooted hypocotyl derived plant after 28 days of culture initiation with a prominent root (arrow). Scale bars **(A,B)**: 1 mm. Scale bar **(C)**: 6 mm.

Although the present study and its derived experiments were focused on *in vitro* shoot organogenesis, some of the cultured explants developed roots instead of shoots. Specifically, 2 weeks after culture initiation, 1.09% of cultured hypocotyls developed vigorous roots with root hairs on the lower zone of the explant (arrow in Figure 5A). The same phenomenon, also located on the proximal part of the explant, was observed in 0.1% of cultured cotyledons 2 weeks after explant culture (arrow in Figure 5B). In another way, spontaneous rooting of *in vitro* regenerants only took place in hypocotyl-derived plants cultured in media without plant growth regulators, where 17.94% of cultured hypocotyls developed shoots on its top and roots in its lower part. After 28 days of culture initiation, hypocotyl-derived plants spontaneously rooted were ready for start the acclimatization process (Figure 5C).

### Acclimatization of *in vitro* Regenerated Plants

**FIGURE 6:**
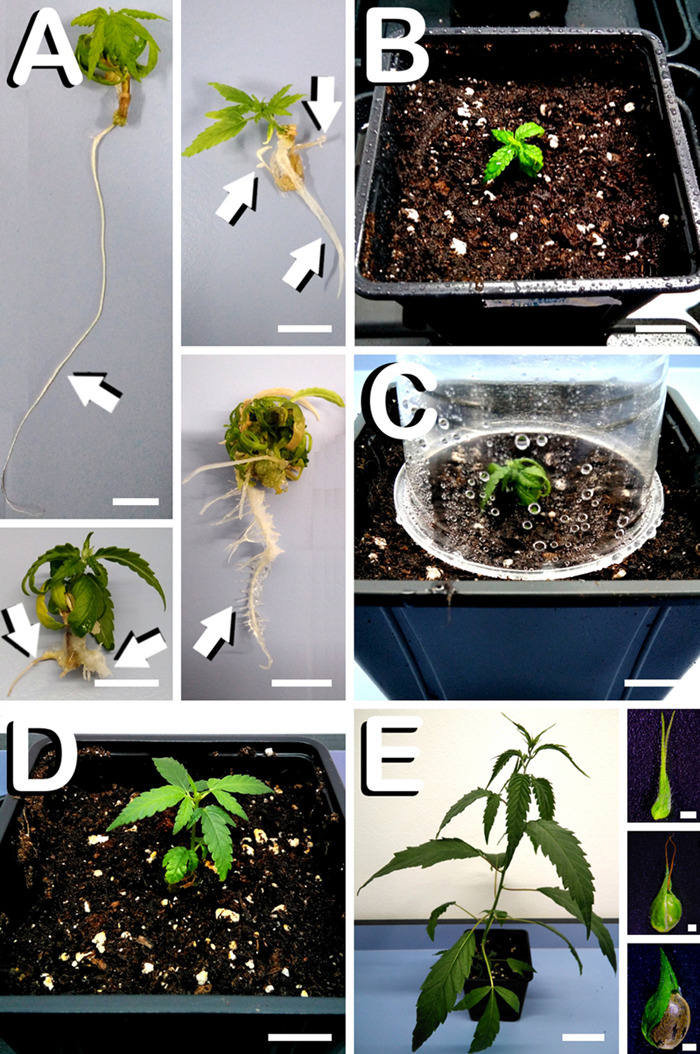
Acclimatization process of hypocotyl derived plants in *C. sativa*. The different developmental stages are described as follows: **(A)** Radicular system of hypocotyl derived plants spontaneously rooted 28 days after culture initiation, where different root morphogenesis patterns can be observed (arrows). **(B)** Small plant just after being transplanted to pots (2 L) with fertilized commercial substrate. **(C)** Plastic vessel covering the *in vitro* regenerated plant in order to avoid desiccation. **(D)** Hypocotyl derived plant exposed to the environmental humidity 6 weeks after culture initiation. **(E)** Female hypocotyl derived hemp plant showing sexual functionality 8 weeks after *in vitro* explant inoculation (insets illustrates, from top to bottom, unfertilized female flower, fertilized female flower during seed formation and mature seed final development). Scale bars **(A–D)**: 12 mm. Scale bar **(E)**: 60 mm. Scale bars of insets **(E)**: 1 mm.

Since only plants regenerated from hypocotyls developed spontaneous rooting, exclusively hypocotyl derived plants were submitted to the acclimatization process. The first step consisted of carefully washing the remaining gellified medium from roots. After 28 days of *in vitro* culture, regenerants showed different root morphogenesis patterns as observed in Figure 6A, where long, medium and short size roots can be visualized, together with a robust main root with a prominent development of secondary roots. Vigorous development of the radicular system guaranteed successful acclimatization of hypocotyl derived plants. At this point, regenerants were ready for transplant in small pots (2 L) with fertilized commercial substrate (Figure 6B), although placement of transparent plastic vessels was necessary to retain humidity and avoid desiccation of plants (Figure 6C). However, after 1 week of progressive exposition of regenerants to the environmental humidity, the acclimatization process ended and hypocotyl-derived plants displayed a vigorous growth, as can be observed in Figure 6D, where a healthy regenerant stands out 6 weeks after culture of hypocotyls. In order to verify the proper development of *in vitro* regenerants, acclimatized plants were grown during two additional weeks and were manually pollinated. As shown in Figure 6E, hypocotyl derived plants showed sexual functionality 8 weeks after *in vitro* explant inoculation, as can be deducted from the fact that female flowers developed viable seeds after manual pollination (insets in Figure 6E). Following this protocol, 100% of hypocotyl-derived plants spontaneously rooted were successfully acclimatized.

### Ploidy Evaluation of Explants and *in vitro* Regenerated Plants of *C. sativa*

**FIGURE 7:**
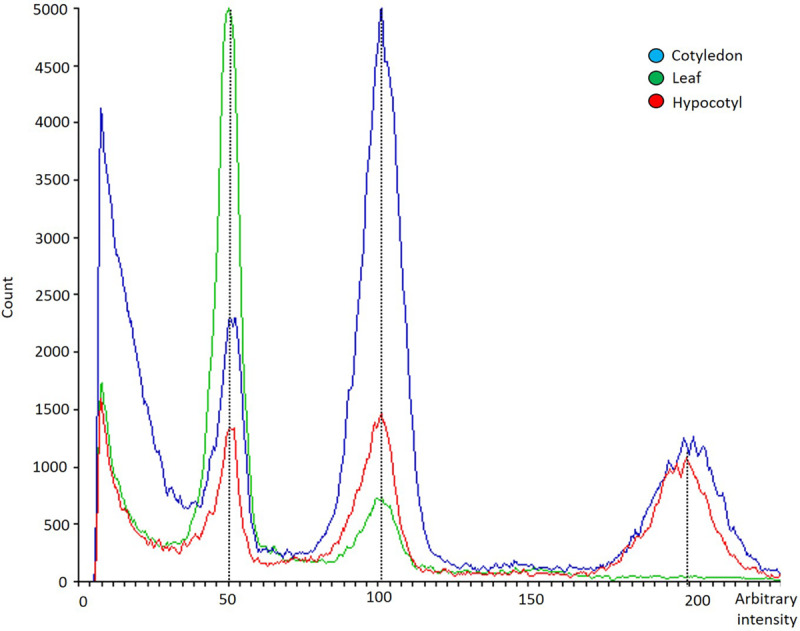
Flow cytometry histogram showing polysomatic pattern in cotyledons (blue), hypocotyls (red) and first pair of true leaves (green) from *C. sativa*. The *x*-axis represents a fluorescence intensity level proportional to the nuclear DNA content. The peak located at the value 50 corresponds to the diploid nuclei in phase G1, the peak located at the value 100 corresponds to the sum of the diploid nuclei in phase G2 and the tetraploid nuclei in phase G1, while the one at the value 200 represents tetraploid nuclei in G2 phase. The *y*-axis indicates the number of nuclei analyzed.

The analysis of the ploidy level of freshly dissected cotyledons, hypocotyls, and true leaves of 7 days-old seedlings of the five varieties evaluated revealed that only true-leaves (green) showed a diploid pattern, while cotyledons (blue) and hypocotyls (red) exhibited a mixoploid pattern (with diploid and tetraploid cells) (Figure 7). All varieties evaluated in this experiment displayed the same polysomatic pattern for the different explants analyzed.

**FIGURE 8:**
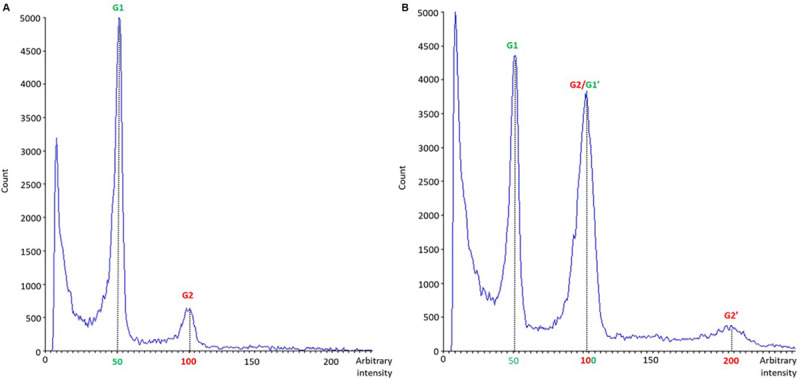
Nuclear DNA histogram patterns of diploid **(A)** and mixoploid **(B)** *in vitro* regenerated plants of *C. sativa* analyzed by flow cytometry. The *x*-axis represents a fluorescence intensity level proportional to the nuclear DNA content. The peak located at the value 50 corresponds to the diploid nuclei in phase G1, the peak located at the value 100 corresponds to the sum of the diploid nuclei in phase G2 and the tetraploid nuclei in phase G1, while the one at the value 200 represents tetraploid nuclei in G2 phase. The *y*-axis indicates the number of nuclei analyzed.

A total of 35 *in vitro* regenerated plants (17 from cotyledons, 15 from hypocotyls and three from leaves) were analyzed by means of flow cytometry 28 days after tissue culture initiation. Only diploid and mixoploid plants (with diploid and tetraploid cells) were obtained. Differences in nuclear DNA histogram patterns between diploid (Figure 8A) and mixoploid (Figure 8B) plants are represented in a flow cytometry histogram (Figure 8). As illustrated in Table 5, no significant differences were identified between cotyledon and hypocotyl-derived plants in terms of ploidy level of *in vitro* regenerants. Cotyledons and hypocotyls produced, respectively, 82.35 and 86.67% of diploid regenerants (Table 5). Regarding mixoploid regenerants, both cotyledons and hypocotyls exhibited a significant capacity to generate them, with, respectively, 17.65 and 13.33% of mixoploid *in vitro* regenerated plants (Table 5).

**TABLE 5:** Effect of explant on ploidy level of *in vitro* regenerated plants coming from cotyledons, hypocotyls and leaves of *C. sativa*.

**2X Regenerants**				**2X + 4X Regenerants**	
**Factor**	**n**	**Diploid regenerants (%)**	**Significance^a^**	**Mixoploid regenerants (%)**	**Significance^a^**
**Explant**					
Cotyledon	17	82.35 ± 9.53	a	17.65 ± 9.53	a
Hypocotyl	15	86.67 ± 9.08	a	13.33 ± 9.08	a
Leaf^*b*^	3	100.00 ± 0.00		0.00 ± 0.00	

*Mean of diploid and mixoploid regenerants (%), significance and sample size (*n*) values are presented in different columns. For each explant, mean of diploid and mixoploid plants is expressed as a percentage (±SE) relative to the total amount of plants submitted to flow cytometry analysis. ^*a*^Different letters among the levels of explant factor indicate significant differences between them (*p* < 0.05) according to non-parametric Kruskal–Wallis and pairwise Wilcoxon tests. ^*b*^Not analyzed statistically.*

## Discussion

### Hypocotyl Presents a High Potential for *in vitro* Direct Regeneration of *C. sativa* Plants Without Addition of Plant Growth Regulators to the Culture Medium

Although significant differences were found for all factors evaluated, the most relevant differences were observed in the type of explant. Specifically, hypocotyl was significantly better than cotyledons and leaves in terms of shoot organogenic potential, as can be concluded from the fact that hypocotyl resulted in more than a 10-fold increase of shoot induction rate in comparison with cotyledon. In contrast, leaves displayed a poor ability to promote shoot organogenesis. On the other hand, significant differences were detected between the different levels of the factor variety, which demonstrates how genotype affects hypocotyl-derived plant regeneration in *C. sativa*, although even the worst performing variety evaluated in hypocotyl experiments reached a high shoot organogenesis rate. Additionally, despite the inherent genetic heterogeneity among individuals of a single *C. sativa* variety (Lata et al., 2016a, 2017), variation between *in vitro* shoot organogenesis rate of hypocotyls coming from individuals of the same variety was relatively low in our experiments. The fact that low values of variation statistics were observed within each variety suggests that intravarietal variation for hypocotyl regeneration capability is limited in *C. sativa*, which consolidates the high potential that this explant present for plant *in vitro* regeneration in this species. Comparing our results with related bibliography, although Wahby et al. (2017) failed to regenerate plants from transformed hemp hypocotyl-derived calli, and Smýkalová et al. (2019) suggested that hypocotyls are not suitable explants for hemp multiplication, lacking of organogenic potential probably due to its low concentration of active cytokinins, it is worth noting that *in vitro* plant regeneration from hypocotyl-derived calli has already been reported in *C. sativa* (Mandolino and Ranalli, 1999; Movahedi et al., 2016a, b). However, in the former works, regeneration rate is not quantified and, probably due to the addition of plant growth regulators in the culture medium, a callus formation phase takes place prior to shoot organogenesis, which may compromise the genetic fidelity of regenerants with respect to the donor plant (Evans and Bravo, 1986; Ramírez-Mosqueda and Iglesias-Andreu, 2015). In this context, it should be noted that, to the best of our knowledge, this is the first report of direct *in vitro* regeneration of *C. sativa* plants from hypocotyls, and the first work in which efficient direct *in vitro* shoot organogenesis is promoted on explants lacking already developed meristems and cultured in medium without plant growth regulators. Since age plays a key role in shoot organogenic potential of explants, as has been described in cotyledons of *C. sativa* (Chaohua et al., 2016), probably in our case, the use of hypocotyls from 7-days-old seedlings with fully expanded first pair of true leaves, together with suppression of plant growth regulators in shoot induction medium, can make the difference with previously published studies concerning hypocotyl derived plant regeneration in *C. sativa*.

Conversely to what occurs with cotyledons, which are unable to regenerate plants in hormone-free medium, our protocol allows a high rate of shoot organogenesis from hypocotyls of all tested varieties without addition of plant growth regulators to the shoot induction medium. *In vitro* regeneration of plants from hypocotyls without using plant growth regulators, has been reported in species like *Capsicum annuum* L. (Ezura et al., 1993) or *Passiflora setacea* D. C. (Vieira et al., 2014). Endogenous hormone levels present in hypocotyls of *C. sativa* could be influencing their ability to generate plants through *in vitro* culture, as can be deducted from the fact that it is possible to promote *in vitro* plant regeneration from hypocotyls even when exogenous supply of plant growth regulators is omitted. In this sense, it has been previously reported how one of the most important factors in adventitious organ formation is the endogenous auxin:cytokinin balance and not the amount of auxin or cytokinin added in a medium (Tanimoto and Harada, 1984). In this respect, it has already been published (Lata et al., 2017, and references therein) the influence exerted by the organ from which explants are dissected (and their endogenous level of plant growth regulators) on shoot organogenesis induction rate. From all the above mentioned facts, it can be concluded that the choice of the primary explant and its related endogenous hormonal levels are crucial for *in vitro* newly meristem formation in *C. sativa*, although more research remains to be done in order to clarify this hypothesis, since Smýkalová et al. (2019), after analyze endogenous hormone levels of hypocotyls from *C. sativa*, reported how endogenous cytokinin concentrations were below the limit of detection.

On the other hand, dissection of explants is another factor which could be influencing *in vitro* shoot organogenesis in hypocotyls coming from *C. sativa*. Since according to common knowledge, cytokinins are produced predominantly in the root meristem and auxins are synthetized in the shoot meristem, and both types of phytohormones can migrate from roots and shoots to their action site through phloem and xylem (Beck, 1996), segmentation of both shoot and root apical meristems as a result of hypocotyl dissection could modify the endogenous hormonal interaction between auxins and cytokinins, leading to an appropriate environment for shoot organogenesis development in hypocotyls.

### Hypocotyl Derived Plants Can Root Spontaneously in Hormone-Free Medium, Being Completely Acclimatized in Only Six Weeks

The same reasoning described above could also explain the fact that, after shoot development in the top of the hypocotyl, auxins produced endogenously in the shoot meristem could promote the spontaneous rooting of *in vitro* regenerants. Regarding this, while *in vitro* spontaneous rooting of regenerated plants has already been reported in other species like *Coleus forskohlii* Briq. (Sairam Reddy et al., 2001), *Cotinus coggygria* Mill. (Metivier et al., 2007) or *Bambusa vulgaris* Schrad. ex J. C. Wendl. (Furlan et al., 2018), its relation with endogenous auxin content of shootlets raised *in vitro* has also been described (Minocha, 1987). On the other hand, root development on the lower portion of hypocotyls of *C. sativa* without apical shoot meristem formation has also been described (LaRue, 1933). In our case, spontaneous rooting of *in vitro* regenerants represents an added advantage of our regeneration protocol, as a separate auxin containing medium is not required for root induction. In any case, although it was necessary to promote rooting of more *in vitro* regenerated plants, there are several published protocols concerning *in vitro* rooting of *C. sativa* shootlets with an efficiency not below 80% (Lata et al., 2009, 2010, 2016a,b; Wang et al., 2009; Chaohua et al., 2016; Parsons et al., 2019).

We found that hypocotyls cultured in medium without plant growth regulators reached the third highest shoot induction rate of the evaluated media without presenting significant differences with the other two media with better percentages of shoot organogenesis. In addition, hypocotyls of all tested varieties were able to develop shoots in this medium. This, coupled with the absence of significant differences in terms of number of shoots per responding explant between the four media with the best shoot induction rates, has led us to suggest the combined use of hypocotyls and hormone-free medium as a suitable combination to obtain hypocotyl derived plants spontaneously rooted and completely acclimatized in just 6 weeks.

### Pericycle Cells Adjacent to Xylem Poles Could Be the Origin of *in vitro* Regenerated Plants of *C. sativa*

In order to infer the possible origin of *in vitro* regenerants from cotyledons, hypocotyls and true leaves coming from 7-days-old seedlings, we examined transversal sections of hypocotyls and, while no presence of already developed meristems or its traces was observed on them, pith, cortex and epidermis were easily identified. Our observations are consistent with those documented by Behr et al. (2016), who studied the development of the secondary tissues in *C. sativa* hypocotyls, illustrating with great detail cross-sections of hypocotyls during different periods after sowing. As presented in their work, cross-sections of hypocotyls 6 and 9 days after sowing are coincident with our studies, since epidermis, cortex and pith can be easily differentiated and their respective anatomy is also concurrent with our findings, which allows us to discard the presence of already developed meristems or its traces in hypocotyls of *C. sativa*, and reject its possible role in the regeneration processes observed in this study.

The fact that the two primordia emerged from the top of hypocotyls were always distributed in the periphery of the organ and aligned one in front of the other, led us to hypothesize that regenerated plants originated always from the same type of cells. In a work by Miller (1959) concerning secondary growth in the root and seedling vascularization of *Humulus lupulus* L., the only species together with *C. sativa* belonging to Cannabaceae family, the internal structure of roots, hypocotyls and cotyledons of seedlings was studied. In this former work, hypocotyl cross-sections drawings detailed the connection between root and cotyledons of the seedling, describing not only the same regions than in our hypocotyl transversal section, but also two protoxylem poles situated in a peripheral position and distributed in opposite sides, whose location strongly resembles the regeneration area of hypocotyl derived meristems in our experiments. Furthermore, Miller (1959) also describes how only one protoxylem pole is located in the median strand of the base of each cotyledon. Since in our study, plant regeneration from cotyledons always was located in the central region of the basal zone of the explant, it is reasonable to hypothesize that cotyledon and hypocotyl derived plant regeneration in *C. sativa* originates from xylem poles. We also found another study that supports this theory, in which xylem cells and its peripheral distribution in two distinctly separated xylem traces distributed in opposite sides, were visualized in hypocotyl transversal sections coming from 9-days-old seedlings of *C. sativa* (Behr et al., 2018). Again, in this preceding work, there is no trace of already developed meristems in hypocotyl cross-sections. With respect to our observations concerning leaf derived plant regeneration, although in this study only five plants were regenerated from leaves, it is remarkably how all of them were originated from leaf-petiole transition zone, as it has been described in species like *Morus indica* L. (Mhatre et al., 1985), previously included together with *C. sativa* in the Moraceae family, or other species like *Beta vulgaris* L. (Detrez et al., 1988) or *Tanacetum cinerariifolium* (Trevir.) Schultz-Bip (Hedayat et al., 2009). The fact that vascularization also takes place in petioles, as it does also on stems, and that leaf regenerated plants always emerged from petioles, could fit with our hypothesis concerning pericycle-derived *in vitro* shoot organogenesis in this species. This extends the scope of our protocol toward micropropagation purposes, adding the possibility to produce multiple clones genetically identical to the elite plants already selected from which they could be derived, although assessment of genetic fidelity by inter simple sequence repeat (ISSR) marker assay is recommended, as it has been performed in other works concerning *C. sativa* micropropagation (Lata et al., 2016a, b). In this respect, it is important to emphasize how responding cotyledon and hypocotyl explants and its derived regenerants, while were maintained in glass-tubes, continued producing multiple shoots even 2 months after culture initiation.

In the line of this hypothesis, Atta et al. (2009) described how pluripotency of *Arabidopsis* xylem pericycle cells is responsible of meristem regeneration from root and hypocotyl explants grown *in vitro*. Finally, it should be noted that Beeckman and De Smet (2014) describe how pericycle cells encircling the xylem pole are considered as an extended meristem which retain the capacity to undergo asymmetric cell division even when other cells have differentiated, and that some pericycle cells surrounded by differentiated cells can still become programmed to begin to proliferate, thus leading to the initiation of a new organ, which could explain how in our work, *in vitro* plant regeneration always developed directly with no need of cell dedifferentiation. Although more research remains to be done in order to validate our hypothesis, the implication of vascular traces on the regenerative capability of the evaluated explants could explain the different shoot organogenesis events observed in our work.

### Polysomaty Is Present in Cotyledons and Hypocotyls of *C. sativa* Seedlings

The term polysomaty was first applied by Langlet (1927) to the condition of those cells in the somatic tissues of a plant which contain multiples of the typical chromosome number (Ervin, 1941), being first described in the early literature by Stomps (1910) in *Spinacia oleracea* L. Since its discovery, polysomaty has been reported in a wide range of species as diverse as *Cucumis melo* L. (Ervin, 1939), *Beta vulgaris* L. (Sliwinska and Lukaszewska, 2005), *Chenopodium quinoa* Willd. (Kolano et al., 2008) or more recently in *Solanum melongena* L. (García-Fortea et al., 2020). Endomitosis or endorreduplication are described as possible causes that may lead to polysomaty (D’Amato, 1964; Bubner et al., 2006), whose occurrence is related with growth and differentiation of tissues. It has also been reported how plant tissues frequently contain a proportion of endopolyploid cells (Ramsay and Kumar, 1990, and references therein) and how portions of the plant such as storage organs and vessels often contain polyploid cells (Adelberg et al., 1993). Concerning polysomaty in *C. sativa*, it should be noted how it was first described in root meristems of the species by Litardière (1925). Langlet (1927) pointed that the doubled number of chromosomes in root meristems coming from *C. sativa* resulted from two successive cleavages of each chromosome during the prophases, while Breslavetz (1926, 1932) proposed nuclear fusion as the cause of the polysomatic condition described in *C. sativa* roots.

Since there was a lack of literature concerning polysomaty in organs other than roots, we analyzed the ploidy level of cotyledons, hypocotyls and true leaves coming from 7-days-old seedlings of *C. sativa* by means of flow cytometry. To our knowledge, this is the first study describing polysomaty in cotyledons and hypocotyls of *C. sativa*. In light of our results, while leaves preserved the diploid pattern typical of the species, cotyledons and hypocotyls displayed a polysomatic pattern containing diploid and tetraploid cells, and therefore should be considered as mixoploid organs. Our findings concerning polysomaty found in cotyledons and hypocotyls of *C. sativa*, open new opportunities such as the development of polyploids through *in vitro* plant regeneration from these organs.

### Mixoploid Plants Can Be Regenerated After *in vitro* Culture of Hypocotyls and Cotyledons Coming From Seedlings of *C. sativa*

Polyploids are associated with enlarged organ sizes, increased biomass yield, phytochemical content and metabolic products, enhanced ability to adapt to biotic and abiotic stresses, and with changes on gene regulation (Van Hieu, 2019). Additionally, development of polyploid plants, in particular tetraploids, could be useful in plant breeding for development of triploid varieties with seedless fruits, as it has been demonstrated in *Citrullus lanatus* (Kihara and Nishiyama, 1947), *Cucumis melo* L. (Adelberg et al., 1993) or in *Citrus* spp. (Recupero et al., 2005). Since polyploid nuclei may sometimes be the progenitors of a cell generation, giving rise to a patch of polyploid tissues (D’Amato, 1952) and after being aware of polysomaty in cotyledons and hypocotyls of *C. sativa*, we evaluated if we had obtained polyploid regenerants which could be useful for cannabinoid production. In this respect, we detected no significant differences between explants in terms of polyploidization of regenerated plants and how cotyledon and hypocotyl were the only explants capable to generate mixoploid plants. It should be noted how polyploidization uses to be associated with enhanced levels of secondary metabolites in a large number of species (Iannicelli et al., 2019), although there are also exceptions, as is the case of *Coffea arabica* L. and *Coffea canephora* L. (Silvarolla et al., 1999), where polyploidization led to decreased levels of caffeine in leaves. In the case of *C. sativa*, results found in the literature are contradictory. While Clarke (1981) found that polyploidization increased THC levels while decreased CBD and cannabinol (CBN) content, and that triploids proved to be inferior to both diploids and tetraploids in terms of cannabinoid production, Parsons et al. (2019) got a similar chemical profile between tetraploids and diploids, although notable increases in CBD and sesquiterpenes were associated with tetraploids. In relation to mixoploids and their cannabinoid content, Mansouri and Bagheri (2017) demonstrated that polyploidization increased significantly the content of THC in mixoploid plants compared with tetraploid and diploid plants, and that tetraploid plants had lower amounts of this substance in comparison with diploids, suggesting that mixoploids could be useful to produce THC for commercial use. Finally, it should be emphasized how polyploidization in *C. sativa* has always been induced by treating apical meristems of the plant with chemical microtubule disruptors with a high toxicity grade, such as colchicine or oryzalin (a less toxic alternative to colchicine), and that chemically induced polyploid plants often revert back to the diploid condition (Clarke, 1981), forcing to test the ploidy level of polyploid plants throughout generations.

## Conclusion

In conclusion, due the high regenerative capacity of hypocotyl and that only hypocotyl-derived *in vitro* regenerants were able to spontaneously rooting, together with the absence of significant differences between media with the best shoot induction rates with respect to number of shoots per responding explant, and between cotyledon and hypocotyl derived plants in terms of polyploidization, we suggest the culture of hypocotyls in hormone-free medium as the most suitable of the treatments evaluated in this study. Our protocol makes direct *in vitro* regeneration of *C. sativa* plants less aleatory and genotype-dependent, so it could have important connotations in exploitation of contemporary plant breeding techniques like genome editing (e.g., by using CRISPR/Cas gene edition) or mutagenesis, being also useful for micropropagation and for the development of polyploid varieties with enhanced levels of cannabinoids without using toxic chemical microtubule disruptors.

## Data Availability Statement

The datasets generated for this study are available on request to the corresponding author.

## Author Contributions

AG-Á, JP, and FH conceived and designed the research. AG-Á and EG-F performed the experiments. All authors analyzed the results, read and approved the manuscript for publication. AG-Á wrote the manuscript and was responsible for verification of the manuscript. EG-F, JP, and FH reviewed and edited the manuscript.

## Conflict of Interest

AG-Á declares that his employer (Ploidy and Genomics Ltd.) was seeking a patent over the protocol presented:

Patent applicant: Ploidy and Genomics Ltd.

Name of inventor: Alberto Galán Ávila

Application number: EP19383210.2

Status of application: Under review

Specific aspect of manuscript covered in patent application: Culture of hypocotyl, cotyledon and/or true leaf explants in the different media described in the manuscript for micropropagation of *Cannabis sativa* L. and obtention of polyploid, mutagenized and/or genome-edited plants.

The remaining authors declare that the research was conducted in the absence of any commercial or financial relationships that could be construed as a potential conflict of interest.
